# Qualitative assessment of proposed visual key information pages for informed consent

**DOI:** 10.1017/cts.2024.662

**Published:** 2024-11-21

**Authors:** Krista E. Cooksey, Eliana Goldstein, Clara Lee, Jessica Mozersky, Kimberly A. Kaphingst, Victor Catalan Gallegos, Mary C. Politi

**Affiliations:** 1 Department of Surgery, Division of Public Health Sciences, Washington University School of Medicine, St. Louis, MO, USA; 2 Department of Surgery, Division of Plastic and Reconstructive Surgery, University of North Carolina at Chapel Hill School of Medicine, Chapel Hill, NC, USA; 3 Department of Medicine, Division of General Medicine & Geriatrics, Washington University School of Medicine, St. Louis, MO, USA; 4 Department of Communication, University of Utah, Salt Lake City, UT, USA; 5 Huntsman Cancer Institute, University of Utah, Salt Lake City, UT, USA

**Keywords:** Informed consent, health literacy, visual design, key information section, Common Rule

## Abstract

**Introduction:**

The 2018 Common Rule revision intended to improve informed consent by recommending a concise key information (KI) section, yet provided little guidance about how to describe KI. We developed innovative, visual KI templates with attention to health literacy and visual design principles. We explored end users’ attitudes, beliefs, and institutional policies that could affect implementing visual KI pages.

**Materials and methods:**

From October 2023 to April 2024, we conducted semi-structured interviews with principal investigators, research staff, and Institutional Review Board (IRB) personnel, including those in oversight/management, and community partners. Forty participants from three academic institutions (in the Midwest, Southeast, and Mountain West) viewed example KI pages and completed interviews. We coded written transcripts inductively and deductively based on the capability, opportunity, and motivation to change behavior (COM-B) framework. Data were analyzed using content analysis and organized thematically.

**Results:**

Participants responded positively to the visual KI examples. They discussed potential benefits, including improving information processing and understanding of study procedures, diversity in research, trust in research, and study workflow. They also described potential challenges to consider before widespread implementation: IRBs’ interpretations of federal guidelines, possible impacts on IRB submission processes, the effort/skill required to develop visuals, and difficulty succinctly communicating study risks. There was no consensus about when to use visual KI during consent, and some wondered if they were feasible for all study types.

**Discussion:**

Visual KI offers a promising solution to long-standing informed consent challenges. Future work can explore resources and training to address challenges and promote widespread use.

## Introduction

Obtaining informed consent is at the cornerstone of conducting ethical research [1]. Although extensive research has identified how to improve participant understanding and engagement in decisions about study participation, participants often lack understanding about studies [1–4]. Despite well-documented strategies to facilitate informed choice such as using plain language, short sentences with fewer words, pictures, and bullet points, these strategies have not been widely translated into routine consent procedures [3].

To help standardize efforts to improve consent processes, the 2018 revision to the Federal Policy for the Protections of Human Subjects (45 CFR 46), or the Common Rule, required consent documents to begin with a “concise and focused” presentation of the key information in a way that “facilitates comprehension” and may help someone “understand the reasons why one might or might not want to participate in the research [5].” The federal Office for Human Research Protections (OHRP) recommends that the key information section include a statement of voluntary participation and right to discontinue participation; purpose of the research; expected duration; procedures; reasonably foreseeable risks and discomforts; reasonably expected benefits; appropriate alternative procedures; and compensation and costs related to participation [6].

Generally, the key information section is meant to ensure that the most important information a “reasonable person” would want to know is contained upfront rather than being buried within a document that contains “pages of tables” and “hundreds of risks [7,8].” This guidance was intended to improve consent processes, but there is no requirement about how to create key information sections of consent forms to meet these goals.

In formative work at a single academic institution in the Midwest, we developed a partnership with the Institutional Review Board (IRB) to resolve recruitment challenges resulting from rapid transitions to virtual recruitment during the COVID-19 pandemic [9]. This work led to procedural changes to minimize both participant and research staff burden (e.g., reducing word count, repetition, and deleting confusing non-required language in text-based consents). It prompted institutional policies that allowed our team to explore and leverage the latitude within the key information section to address participant misunderstandings about study details.

As a result of this ongoing partnership, our team recognized a unique opportunity to meet federal guidelines for key information while presenting study information in an innovative, visual way with attention to health literacy and visual design principles. The focus of this current work explores the perspectives of a diverse set of end users, including principal investigators, IRB personnel, research staff, and community partners on a series of proposed visual key information templates. We discuss relevant themes and factors that end users describe as important considerations for implementing visual key information pages into routine practice.

## Materials and methods

### Study design

**Figure 1.**
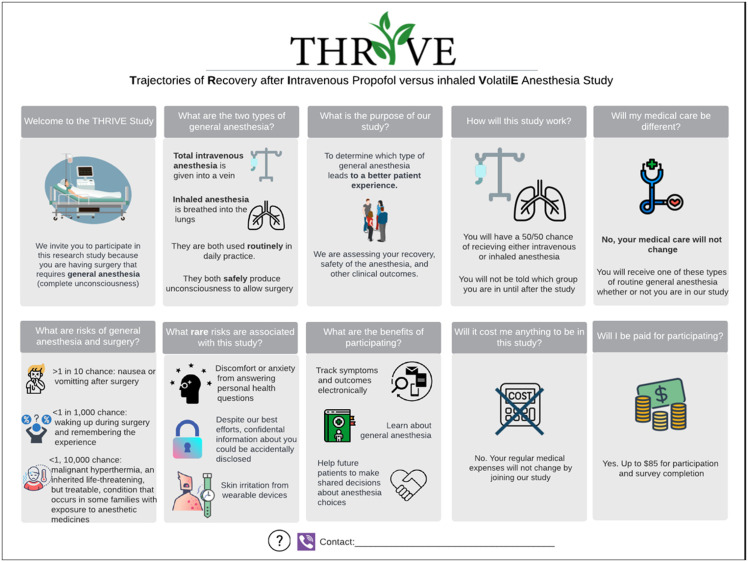
Example 1 displayed during qualitative interviews.

**Figure 2.**
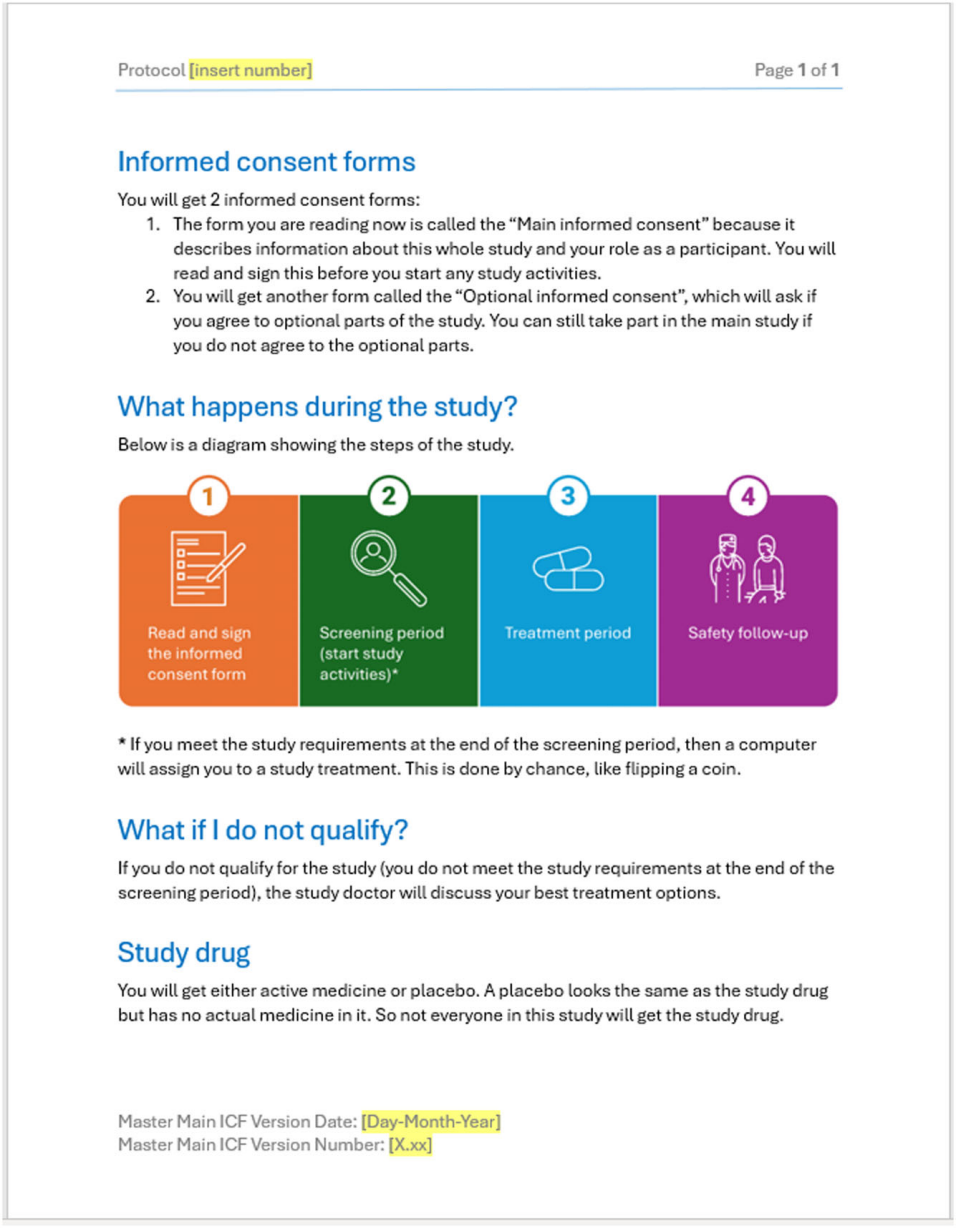
Page from example 2 displayed during qualitative interviews.

**Figure 3.**
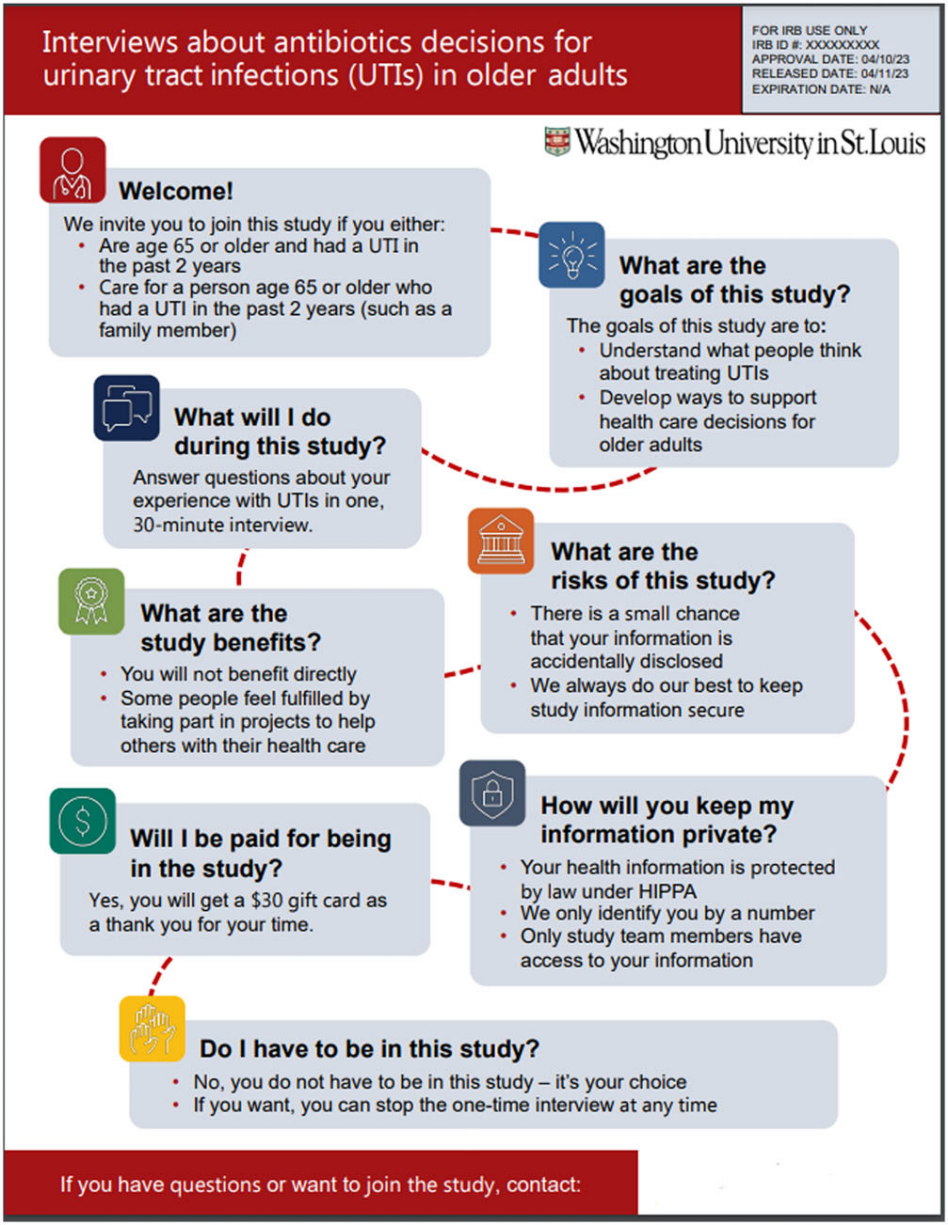
Example 3 displayed during qualitative interviews.

**Figure 4.**
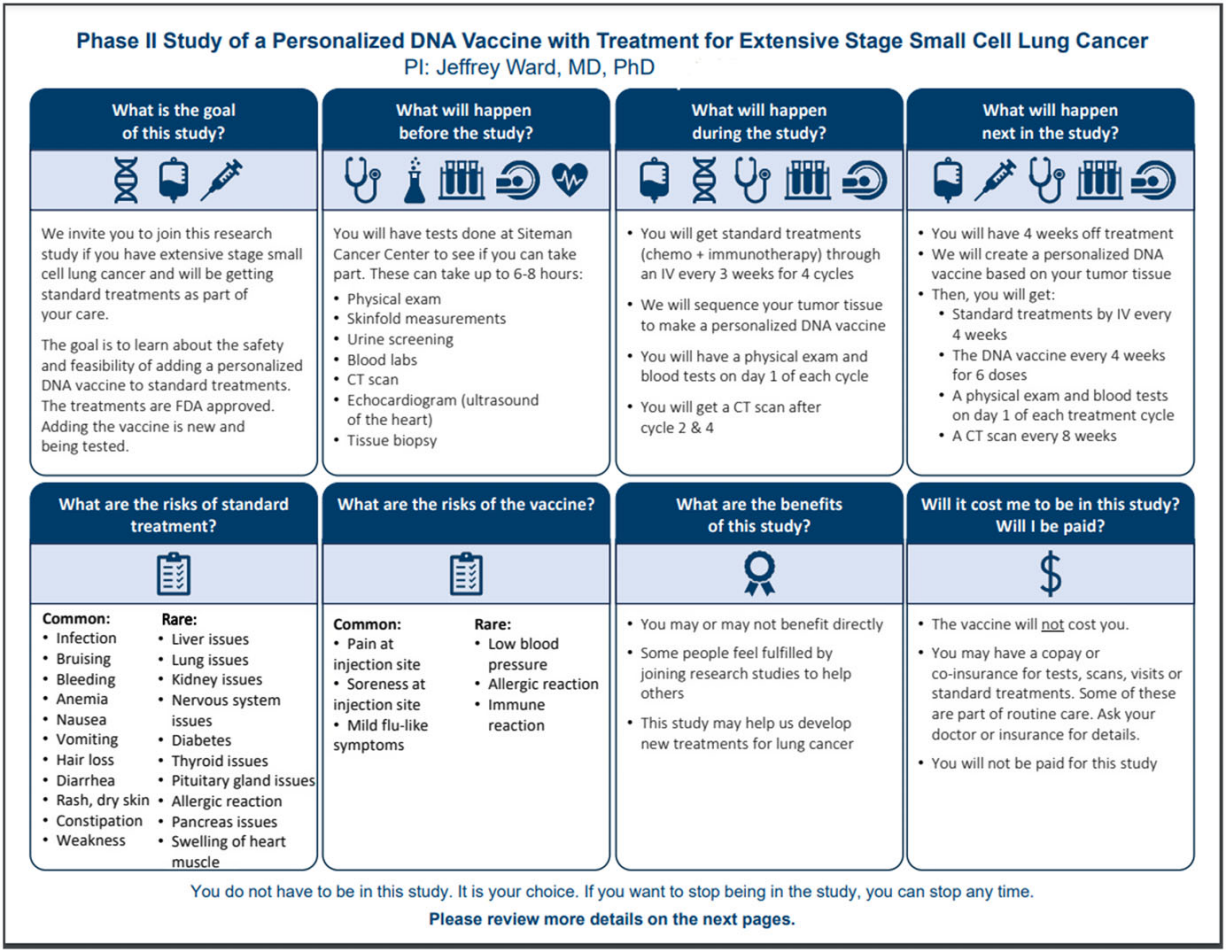
Example 4 displayed during qualitative interviews.

We conducted semi-structured individual interviews from October 2023 to April 2024 via video conference. Participants reviewed four example consent documents that used visual elements to communicate study details (Figures 1–4). Washington University’s IRB approved this project as exempt research (IRB# 202308006).

### Visual design

Visual key information pages were designed in Microsoft PowerPoint to ensure accessibility and minimize costs for specialized design programs. Two visual key information pages were designed by our team in conjunction with principal investigators and research staff. The other two examples were designed through a partnership with Health Literacy Media, a non-profit health communications firm, with support and input from our team and research staff. Special attention was made to plain language and health literacy informed design principles such as increasing white space, shortening sentences, using bullets and easily readable fonts, and avoiding red/green colors [3,10].

One of the key information pages was for an exempt interview study, while the other two were for investigational clinical trials. The principal investigators or institutional leaders suggested these trials for testing. The fourth example was a multi-page master platform consent template, shared by Health Literacy Media, which incorporated icons and other formatting elements throughout a more traditional text-based consent. All were designed for adult (18+ years) participant populations.

Icons and images were created by Health Literacy media or adapted from widely available institutional resources or free online images [11].

### Participants

We recruited participants from three academic centers from diverse regions in the USA. Participants included principal investigators, research staff, IRB personnel (members, leadership, and staff), and community partners (community members, patient advocates, members of community IRBs, and institutional staff involved in community engagement). Initial recruitment was conducted based on publicly available contact information for relevant individuals provided by lead investigators at the three participating sites. Additional participants were identified via snowball sampling and word-of-mouth referrals. Eligible and interested participants provided verbal consent prior to beginning the interviews.

### Procedures

Study staff trained in qualitative interviewing conducted all interviews under the supervision of the principal investigator. Interviews were conducted via video conference and were audio-recorded with participants’ consent. The four templates were sent to participants in advance of their scheduled interview. If the participant did not have an opportunity to review before the interview, they were given additional time prior to the start of the interview.

The interview guide (Appendix A) elicited opinions on benefits and downsides of the visual key information templates, feedback on each example, and recommendations for using visual key information pages during consent (e.g., timing and format of delivery).

Participants received a $30 gift card for completing the interview. Audio recordings were transcribed verbatim. De-identified transcripts were coded by two trained coders (KC and EG) using inductive and deductive coding based on the behavior change wheel, or the capability, opportunity, and motivation to change behavior (COM-B) model of behavior change [12], which posits three factors contributing to adoption of a new behavior: capability, opportunity, and motivation. For this study, we defined the new behavior as adopting visual key information pages. Capability is defined as the individual’s capacity to participate in the behavior (i.e., understanding the purpose of the key information section or its flexibility), opportunity is defined as the external factors that make execution of a behavior possible (i.e., IRB or funder regulations), and motivation includes internal cognitive processes that direct and inspire the behavior (i.e., improving participant understanding or reducing burden) [12].

The first five transcripts were coded by both coders to refine the codebook and reach inter-rater reliability (Cohen’s kappa > 0.75; > 95% agreement). Coders then coded the remaining transcripts individually, meeting regularly to ensure continued agreement. A fidelity check was completed midway through coding. Coders resolved any coding discrepancies with a third team member (MP). Data were analyzed using a content analysis approach and organized into overarching themes.

## Results

### Participants

The research team contacted 88 possible participants involved in the planning, conduct, and/or oversight of human subjects research. Sixty-five percent (*n* = 26) of participants were not directly known by study investigators and were found via public search or identified through snowball sampling. Forty-five percent (*n* = 40) of those contacted were enrolled: 15 research staff, 11 IRB personnel, 10 principal investigators, and 14 community partners. Participants could choose more than one role. Table 1 displays additional participant characteristics. Interviews lasted about 30 minutes.

**Table 1.** Participant demographics (*N* = 40)

**Age**	
Mean (SD)	48.55 (13.75)
Range	29–77
**Gender**	
Female	34 (85.00%)
Male	6 (15.00%)
Non-binary	0 (0.00%)
Prefer not to say	0 (0.00%)
Prefer to self-describe	0 (0.00%)
**Ethnicity**	
Latino/a/Latine or Hispanic	2 (5.00%)
Not Latino/a/Latine or Hispanic	38 (95.00%)
**Race**	
White	34 (85.00%)
Black or African–American	4 (10.00%)
Asian	1 (2.50%)
Native American or Alaskan Native	0 (0.00%)
Native Hawaiian or other Pacific Islander	0 (0.00%)
Not specified	1 (2.50%)
**Institutional role (could select > 1)**	
Principal Investigator (PI)	10 (25.00%)
IRB personnel	11 (27.50%)
Research staff	15 (37.50%)
Community partner	14 (35.00%)
**Length of time in current position**	
Less than one year	3 (7.50%)
1–2 years	7 (17.50%)
3–4 years	7 (17.50%)
5 or more years	23 (57.50%)

### Themes

Several themes emerged from the interviews, including the benefits of using visual key information pages, possible implementation challenges, uncertainty about the best time to use visual key information pages, and uncertainty about their appropriateness for all study types.

#### Theme 1: Participants described numerous potential benefits to using visual key information pages for informed consent

##### a. Information processing and understanding of complex information

Many participants noted that visual key information pages were clearer and less overwhelming compared to text-heavy traditional consent forms:

*“I like it. I mean, I think it’s broken down in just a nice way just to make it a little less overwhelming for participants. Because their brain can have a break in between the sections… [and] the pictures next to it…give the brain a sense to process what they’re going to be reading about.” (P03, Research Staff)*

Others noted that increased understanding through visual displays could promote informed consent:

*“They may actually even understand what they’re consenting to more appropriately than just being read a document or having to read a really long document. That this could help break things down for them so…it was actually informed consent.” (P19, Research Staff)*

***“*** *It wouldn’t surprise me if that would improve accrual rates and ideally, help them to feel and actually be more informed about what they’re consenting to.” (P02, Research Staff)*

Similarly, IRB members stated that visual key information pages were more engaging and digestible compared to traditional consent documents:

*“I would think patients and participants would be more likely to look at these graphic versions or these just nontraditional versions a second time or a third time, and they’re able to fully scan the information more quickly, more efficiently than the text-based version that we normally have.” (P11, PI & IRB Personnel)*

*“…not having to look through a document that feels like a contract…this just feels much more…interactive.” (P40, IRB Personnel)*

*“Making it in these graphics and these boxes makes it a little easier for people to pinpoint exactly what’s going on and follow a logical order.” (P08, IRB Personnel)*

##### b. Improving trust in research

Some participants discussed the potential for visual key information pages to improve trust in research by demystifying studies through clear, comprehendible visuals:

*“I do think it might help those that would be wary of going into a clinical trial or voluntarily choosing to participate in one.” (P13, Research Staff)*

*“I think that people just need to feel more comfortable with science and research in general, and I think this could help bridge that gap.” (P19, Research Staff)*

Others agreed, stating that visuals could help address misconceptions about participation in research:

*“Because I think there’s so many people out there that have a misconception about clinical trials in general. If you can give them something short and sweet with pictures that explains a clinical trial and what is it and that they can get out of it anytime they want to, what the purpose is, I think that would offer a better understanding.” (P35, Community Partner)*

##### c. Inclusivity of participants from diverse backgrounds

Many participants underscored the potential advantage of visuals to address gaps in diversity, equity, and inclusion in research. They most often commented that visuals could possibly reduce language and cultural barriers during recruitment:

*“I think it’s just to motivate people, members of all communities, to be part of research…I know one of the reasons sometimes they’re not participating is because of a language barrier.” (P21, IRB Personnel & Community Partner)*

*“I think that’s why the visuals are nice because visuals can be consistent across different translations.” (P26, Community Partner)*

*“Pictures and Graphics Are Kinda Universal [across Languages].” (P35, Community Partner)*

*“The other advantage…is if you’re translating this… That’s important for diversity in subject populations.” (P29, IRB Personnel)*

##### d. Improving research staff workflow and saving time during consent

Participants discussed how visual key information pages could promote efficiency during the recruitment and consenting process, including for staff.

*“I think it’s going to be easier to interpret and simpler, not only for the patient, but for the study coordinator who is helping to consent the patient. I think it’s going to make their job a little bit easier as well.” (P03, Research Staff)*

A community partner noted that potential participants could quickly assess if they were interested in joining a trial when viewing visuals, which could save time:

*“They see this and go “I don’t want to participate. It may be something, they see the risks and they’re like, ooh, no, never mind. Then you have not wasted a lot of time going over the entire consent, right?” (P31, Community Partner)*

IRB personnel agreed that these could save time during consent:

*“It saves the coordinator time because they’re not sitting down with somebody who then decides not to do it.” (P40, IRB Personnel)*

*“What will happen is study teams, first of all, instead of spending two hours consenting one subject, you spent five minutes consenting me. That’s hours back. Multiply that by all the studies.” (P29, IRB Personnel)*

#### Theme 2: Some participants identified challenges that needed to be considered in order to make visual key information pages part of routine practice

Although most participants were enthusiastic about using visual key information pages as part of informed consent, some expressed potential implementation challenges.

##### a. Uncertainty about IRB acceptance of a visual key information page

Principal investigators, IRB members, and research staff expressed uncertainty about how IRBs would respond to visual key information pages based on institutional goals, norms, and priorities:

*“The IRB is going to say no [to using visual key information pages]…” (P12, PI & IRB Personnel)*

*“I think convincing our <Board> that this is a good template could be challenging because they’re very conservative in the way that they interpret [federal] regulations at our site versus many other university academic centers.” (P03, Research Staff)*

##### b. Possible impact of adding visuals to the IRB review and submission process

Some participants feared that adding visual key information pages would contribute to longer, more complex IRB submission processes:

*“I do think it would probably add potentially a little bit more time to the actual review process because you’re just literally adding another page.” (P04, Research Staff)*

IRB members similarly commented on the technical difficulty of reviewing visual key information pages, which could lengthen review times:

*“There’s a lot of things that look like they’d be really good and participant-centered, and I’d love to approve them, and I think they should be approved, well, but just getting them approved and getting them reviewed is just technically more difficult, which is just a separate issue.” (P17, IRB Personnel)*

##### c. Amount of skill and effort needed to develop visual key information pages

Some participants noted the level of technical skill that may be required to create participant-friendly, esthetically pleasing key information pages. They often discussed the potential burden of time and effort required to develop visual key information pages, which may cause some to revert to traditional text-based templates:

*“I think some study teams feel like the time and attention it would take to create something like this is beyond what they have time and attention for…They’ve already got consent forms that are written just as text that they’re like, well, this is what we know. This is what’s going to be the fastest for us, so this is what we’re going to do. That, I think, is a barrier. I would love to see people overcome that barrier. (P12, PI & IRB Personnel)*

*“From a researcher, I think the barrier would be the creating of this. This requires a set of skills…it’s a new territory.” (P23, PI)*

##### d. How to define the “most important” risk information to include in the visual key information page

Participants communicated different preferences for displaying risks in visual key information pages. While some participants erred on the side of including all possible risks for transparency, others maintained that it was important to highlight main or most study risks upfront.

*“I just think that in looking at them, the thing that is the longest portion of the written consent document tends to be the risks section, but this document sums up those …pages in one bubble or two bubbles. We need to make sure that the patient is fully aware of the risks. We do not want to scare them because I always say before I even start going through those…pages, know that this does not mean you’re going to experience all of these things.” (P22, Research Staff)*

*“I think having a visual representation up front to highlight, “Okay, we’re going to give you this list of 75 different risks, but really, what you’re looking at is you might…experience nausea or vomiting. Those are the main risks.” I think that helps people to separate the wheat from the chaff a little bit.” (P02, Research Staff)*

##### e. Training and resources are needed for research teams and IRBs

Participants expressed that IRB personnel and research teams would both need guidance and training to develop and evaluate visual key information pages.

*“…Honestly…probably the only concern that I would have would be making sure that the IRB reviewers were all on board and trained that this is acceptable and making sure that, as a research staff who would be putting something like this together, that I understood also what had to be there and what was optional….” (P01, Research Staff)*

*“I think for there to be a successful implementation of this new format, there would have to be some standards, and some training done of IRB staff so that they know what to evaluate.” (P23, PI)*

In addition to guidance at the regulatory level, participants emphasized the need for guidance at the research team level to ensure that visual key information pages were effectively developed and followed approved guidelines.

*“Just to make everything smoother…I would go with…some extra training around when you submit one of these or edit one of these, here are the special procedures for this, at least at this institution.” (P17, IRB Personnel)*

*“I think the idea of having your group or some group having some sort of a standardized template that someone’s already done all the work for the most part, and then others can benefit off of that.” (P14, Research Staff)*

#### Theme 3: Participants had mixed opinions about when to use visual key information pages

Some participants thought that the best time to present visual key information pages was before the consent process to give patients an introduction to the study:

*“You’ll go through the whole form with them, but this allows them to ahead of time they can get a better understanding before you even have that thorough conversation with them.” (P07, Research Staff)*

*“I would present this pretty early on in the process, essentially contacting the participant. Let’s say they’re interested in the study, so this is what I would use initially to introduce the project, explain goals of the study, background, what happens, risks, benefits, compensation, all of those things.” (P16, PI)*

Others thought that using visual key information pages during the consent process could help supplement information in the main consent:

*“Then also use it when you’re consenting people. That’s a good document to have. Then you can go through, if it does need a 10-page consent, they can have that as the cliff notes or the cheat sheet as they and the study coordinator go through the full consent. They can make their notes on it or star it or highlight it.” (P29, IRB Personnel)*

*“…I can see this being a first page of a stapled physical consent document if you’re seeing somebody in person.” (P01, Research Staff)*

A few participants thought that the best time to present visual key information pages was after the consent process to serve as a helpful reference tool:

*“If it were me consenting a patient, I would probably do the full consent first. Just say, “Hey, this was a lot,” …I also have this sheet that breaks it down easily that you can quick reference, too,“ that type of situation.” (P03, Research Staff)*

*“I could envision being our roadmap for participants especially in a longitudinal study or something with multiple follow ups. Having that laid out somewhere that’s really easy to look at and understand, “Okay, for the participant this is where I’m at in the project and this is what I can expect moving forward.” Just having a one-pager that they could refer to throughout the study I think would be useful for a lot of people.” (P16, PI)*

#### Theme 4: Participants expressed uncertainty about the appropriateness of visual key information pages for all study types (i.e., exempt and non-exempt studies)

Some participants stated that all study types, exempt and non-exempt, could benefit from using visual consents:

*“I think all trials could benefit from this. It can be actual clinical. It could be translational science, could be observational.” (P36, Community Partner)*

In fact, some participants even noted that of all the study types, complex studies specifically were a perfect opportunity to condense information in a visual summary:

*“I think that there would be some people who would say, “Oh, if the study’s too complicated, you’re not going to be able to put something together like this, that is a good summary.” I would argue it’s for complicated studies where we need a good summary. I think I would argue that complicated clinical trials or studies with lots of visits, lots of procedures, I would argue that this is actually more helpful to those studies.” (P12, PI & IRB Personnel)*

*“Treatment trials are the most trying, I guess, and probably where we have the most fallout or resistance in people enrolling. I think simplifying the information that those patients get is of utmost importance.” (P35, Community Partner)*

However, other participants questioned whether visual summaries were appropriate for non-exempt, complex trials due to uncertainty about how to effectively display all of the required study information:

*“Maybe for an interventional study, you actually just cannot get enough information on something with this format.” (P28, PI)*

*“If you have a study that is super complicated. Maybe a patient wouldn’t necessarily get all the information they need.” (P06, Research Staff)*

*“If the study gets too complex, the template itself might not have the flexibility to replace the key information template, because you might need more in the key information template than would fit comfortably.” (P17, IRB Personnel)*

### Visual key information page recommendations

In response to data collected from these interviews and concurrent feedback from ongoing engagement with additional end users, we compiled a list of recommendations for the development of visual key information pages. Recommendations include: 1) follow OHRP guidelines [6] for what to include in key information pages (i.e., statement of voluntary participation and right to discontinue participation; purpose of the research; expected duration; procedures; reasonably foreseeable risks and discomforts; reasonably expected benefits; appropriate alternatives; compensation and costs related to participation); 2) incorporate institutional needs and preferences; 3) break up sections clearly; 4) follow health literacy design principles [3,10] (i.e., plain language, concise sentences, bullets, and icons); 5) use a vertically oriented design to integrate into full consent documents that are also vertically oriented; 6) aim to keep the visual key information to one page; 7) use consistent colors and icons, preferably from freely available resources to enhance implementation potential; and 8) leave space for an IRB stamp in the appropriate location for one’s institution.

**Figure 5.**
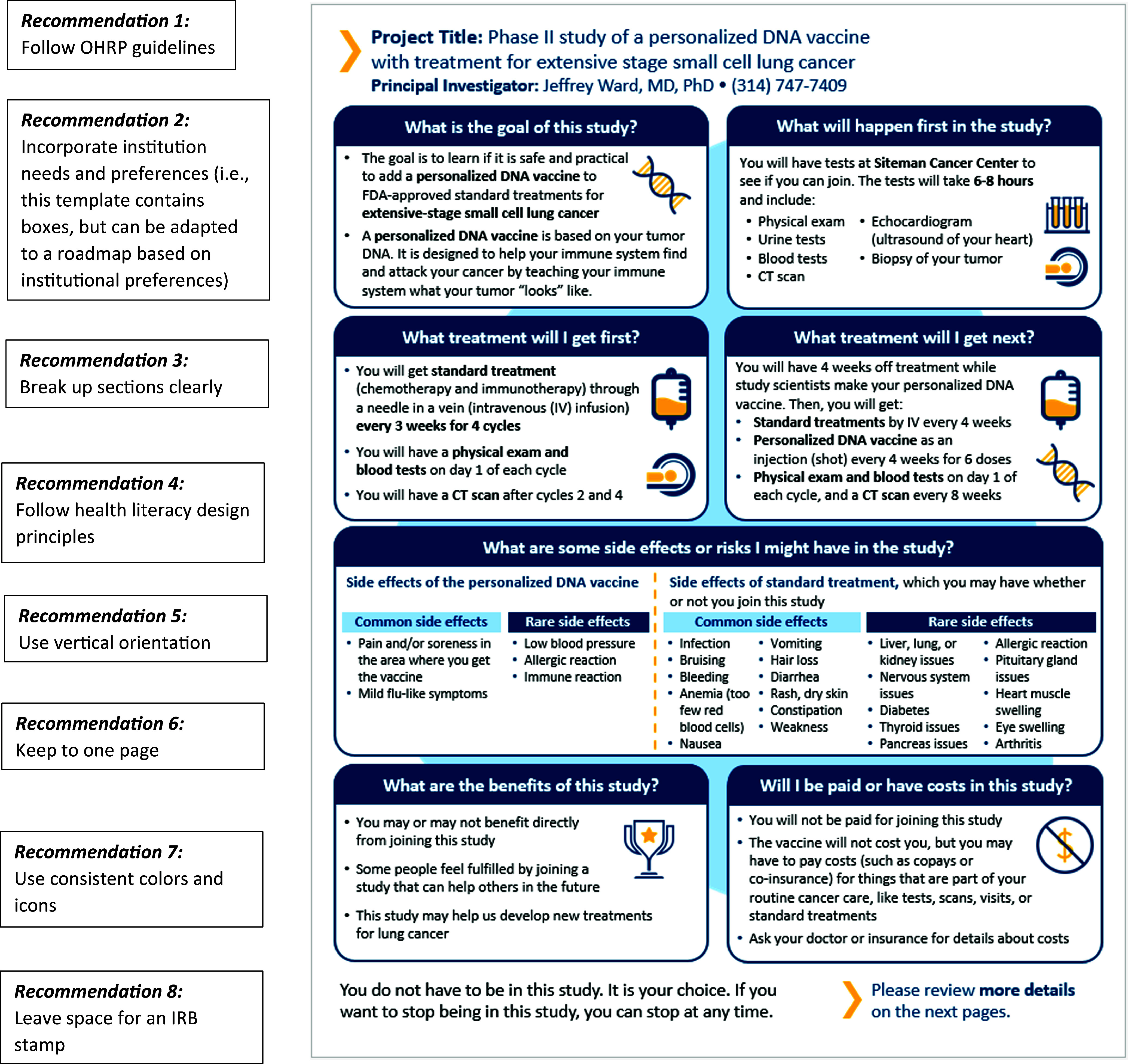
Example of visual key information page revised after interviews following recommendations.

Using these recommendations alongside ongoing engagement with the three IRBs, we revised the visual key information page template. Figure 5 displays one example of updates made to Figure 4 following these suggestions.

## Discussion

Visual key information pages have several potential benefits for participants and research team members. Potential benefits include improved, more efficient information processing and understanding of study materials, addressing mistrust, and promoting inclusivity and diversity in study recruitment, especially clinical trials. Challenges discussed included uncertainty of IRB acceptance, the amount of effort that may be required to create visual consents, when to administer the visuals, the difficulty of effectively condensing complex information, more complicated submission processes, how to best communicate risks, and the appropriateness of using visuals for all study types, especially non-exempt studies.

### Perceptions about IRBs and change

Participants frequently described if visual key information pages would be acceptable to IRBs as a potentially significant implementation challenge. Although many participants favored the idea of visual key information pages, some noted that their IRB was more conservative in their interpretation of federal guidelines [13], which limited perceptions of feasibility of widespread adoption. These attitudes reflect a historic adversarial relationship between research teams and IRBs [14,15]. However, IRB members in this study were overall supportive of using visual key information and provided extensive feedback to ensure that the visual template met all existing guidelines. In fact, some IRB personnel considered how to encourage teams to “step outside of their own comfort zones” and communicate to research teams that they should not be afraid to propose new ideas, like visual key information pages. Future work may explore misconceptions about IRB and research team dynamics and identify strategies to improve attitudes among researchers and IRB members about capacity to change.

### Potential implications for diversity, equity, and inclusion

Many participants noted the positive benefits visual key information pages may have on diversity, equity, and inclusion in research. Inclusion of visuals may help address historic mistrust of research by promoting transparency and demystifying studies through clear, comprehendible visuals with accessible language [16]. Future work will explore translating visual key information pages into additional languages to further support their use in this context.

### Training and resources are needed

Findings also suggest that IRB and research teams would be receptive to additional training and resources to address challenges and help answer remaining questions about how to effectively implement visual key information pages. Ensuring that various end users, especially IRB personnel, understand that visual key information pages can meet federal guidelines was cited as a key component of implementation. Additionally, participants mentioned resources and training were needed for research teams. Training opportunities for research teams include how to develop visual key information pages, tailor templates to meet study specific needs, guidance on when to use visual key information pages during the consent process, and procedures to submit to the IRB. Building upon existing infrastructure, developing resources, and identifying training needs to implement visual key information pages will be an iterative process.

### Opportunities for future prototypes

The flexibility and adaptability of visual key information pages offer additional opportunities to fit the needs of a wide range of study types and study populations. Future work may also explore appropriateness in pediatric studies, which often require assent. Additionally, participants discussed the possibility of incorporating interactive elements into the visual key information pages for virtual/web-based studies. This process could directly link the visual key information page to elements in the main consent document.

## Limitations

Findings should be interpreted within the context of some study limitations. First, findings reflect opinions about the benefits and challenges of using visual key information rather than actual experiences or barriers encountered when implementing them. Empirical data is needed to assess the impact of visual key information on issues such as enhancing diverse recruitment, or lengthening IRB review times. Second, our findings reflect the experiences and opinions of a subset of end users who might have similar characteristics and perspectives given the qualitative research methods and use of snowball sampling or word-of-mouth referrals. In addition, this research was conducted at three institutions with Clinical and Translational Science Awards (CTSA) programs. Because the goal of the CTSA programs are to support innovation in research processes, it is possible that non-CTSA institutions may have different experiences and infrastructure needs affecting implementation of visual key information pages. Finally, the selected COM-B framework may not be relevant for all end users. We asked participants to consider what they thought about their institution, including IRB and research staff, using visual key information pages. However, some community partners (i.e., community members) may not be involved in the decision to adopt the visuals. Despite these limitations, results represent the opinions of a diverse set of key end users (PIs, research staff, IRB personnel, and community partners). The qualitative exploration and in-depth perspectives elicited can serve as a basis for additional empirical research on this topic.

## Conclusion

Visual key information pages offer a promising solution to long-standing informed consent challenges caused by lengthy, complex documents filled with technical and legal language. Pilot studies are underway to gather preliminary data in exempt and non-exempt studies to evaluate the effectiveness of visual key information pages in improving participant knowledge of study procedures and decisional conflict about joining clinical trials. Additionally, current work involves 1) ongoing engagement with Health Literacy Media to develop templates and icon libraries using widely available resources for investigators’ future use; 2) allotting time and resources to ensure that procedures are in place for widespread adoption; 3) revising visual key information templates with ongoing support from the IRBs; and 4) evaluating the usability, acceptability, and feasibility of a proposed toolkit to standardize the development process and aggregate existing design principles, plain language, and readability resources. Future work will gather data on the impact of using visual key information pages on participant-centered outcomes in a randomized controlled trial [17].

## Supporting information

Cooksey et al. supplementary material Cooksey et al. supplementary material

## Supplementary material

The supplementary material for this article can be found at https://doi.org/10.1017/cts.2024.662.

## References

[1] Kadam RA. Informed consent process: a step further towards making it meaningful!. Perspect Clin Res. 2017;8:107–112. doi: 10.4103/picr.PICR_147_16. [published Online First: 2017/08/23]. 28828304 PMC5543760

[2] Nishimura A , Carey J , Erwin PJ , Tilburt JC , Murad M H , McCormick JB. Improving understanding in the research informed consent process: a systematic review of 54 interventions tested in randomized control trials. BMC Med Ethics. 2013;14:28. doi: 10.1186/1472-6939-14-28. 23879694 PMC3733934

[3] Kim EJ , Kim SH. Simplification improves understanding of informed consent information in clinical trials regardless of health literacy level. Clin Trials 2015;12:232–236. doi: 10.1177/1740774515571139. [published Online First: 2015/02/24]. 25701156

[4] Foe G , Larson EL. Reading level and comprehension of research consent forms: an integrative review. J Empir Res Hum Res. 2016;11:31–46. doi: 10.1177/1556264616637483. 27106889

[5] 45 CFR 46. Office for Human Research Protections, 2021. (https://www.hhs.gov/ohrp/regulations-and-policy/regulations/45-cfr-46/index.html). Accessed August 7, 2024.

[6] U.S. Department of Health and Human Services. Draft guidance on key information to facilitate understanding of informed consent. Office for Human Research Protections. U.S. Department of Health and Human Services. (https://www.hhs.gov/ohrp/regulations-and-policy/requests-for-comments/draft-guidance-key-information-facilitating-understanding-informed-consent/index.html). Accessed August 10, 2024.

[7] Mozersky J , Wroblewski MP , Solomon ED , DuBois JM. How are US institutions implementing the new key information requirement? J Clin Transl Sci. 2020;4:365–369. doi: 10.1017/cts.2020.1. [published Online First: 2020/11/28]. 33244420 PMC7681124

[8] Menikoff J , Kaneshiro J , Pritchard I. The common rule, updated. N Engl J Med. 2017;376:613–615. doi: 10.1056/NEJMp1700736. [published Online First: 2017/01/20]. 28103146

[9] Cooksey KE , Mozersky J , DuBois J , Kuroki L , Marx CM , Politi MC. Challenges and possible solutions to adapting to virtual recruitment: lessons learned from a survey study during the Covid-19 pandemic. Ethics & Human Research. 2022;44:23–31. doi: 10.1002/eahr.500148. 36316973 PMC9631333

[10] Jefford M , Moore R. Improvement of informed consent and the quality of consent documents. Lancet Oncol. 2008;9:485–493. doi: 10.1016/s1470-2045(08)70128-1. [published Online First: 2008/05/03]. 18452859

[11] Washington University in St. Louis. Icon library. Washington University in St. Louis. (https://marcomm.washu.edu/icon-library/). Accessed August 10, 2024.

[12] Michie S , van Stralen MM , West R. The behaviour change wheel: a new method for characterising and designing behaviour change interventions. Implement Sci. 2011;6:42. doi: 10.1186/1748-5908-6-42. 21513547 PMC3096582

[13] Larson E , Bratts T , Zwanziger J , Stone P. A survey of IRB process in 68 U.S. hospitals. J Nurs Scholarsh. 2004;36:260–264. doi: 10.1111/j.1547-5069.2004.04047.x. [published Online First: 2004/10/22]. 15495496

[14] Domenech Rodríguez MM , Corralejo SM , Vouvalis N , Mirly AK. Institutional review board: ally not adversary. Psi Chi J of Psychol Res. 2017;22:76–84. doi: 10.24839/2325-7342.JN22.2.76.

[15] Keith-Spiegel P , Koocher GP. The IRB paradox: could the protectors also encourage deceit? Ethics Behav. 2005;15:339–349. doi: 10.1207/s15327019eb1504_5. [published Online First: 2006/04/04]. 16578924

[16] Veluri S, Rastgar Y, Chen J, Doppalapudi N. Creating space for diversity in informed consent forms. HPHR. 2024;84. doi: 10.54111/0001/FFFF3.

[17] National Institutes of Health. Project details: 10917307. NIH Research Portfolio Online Reporting Tools (RePORT). (https://reporter.nih.gov/search/cAUzVARKXkKJi9uKkmyJlw/project-details/10917307). Accessed August 8, 2024.

