# Supplementary figures and images for: Qualitative assessment of proposed visual key information pages for informed consent
Source: J Clin Transl Sci. 2024 Nov 21;8(1):e218. doi: 10.1017/cts.2024.662 (PMC11713442; doi:10.1017/cts.2024.662)

Appendix A: Interview Guide


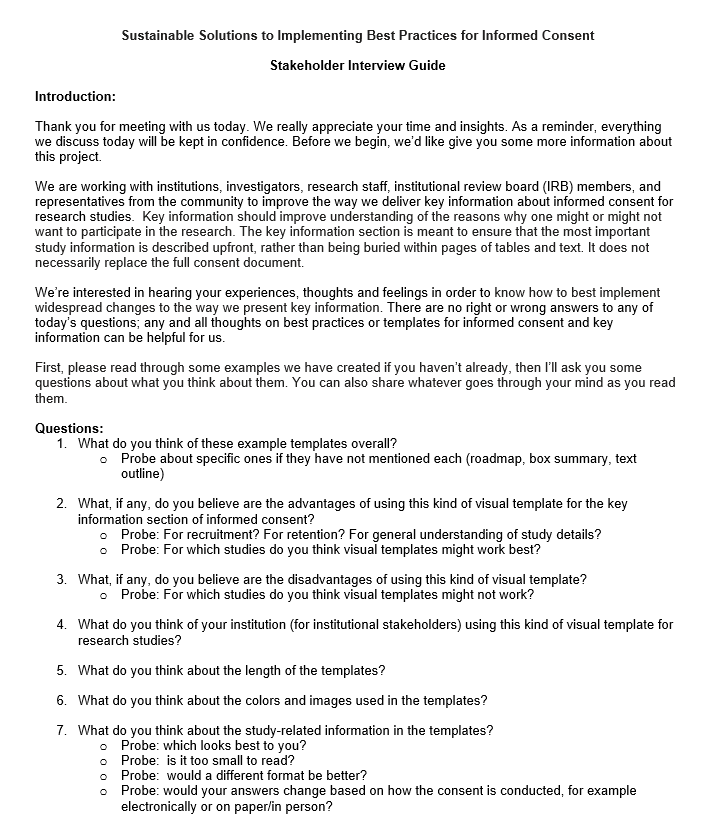


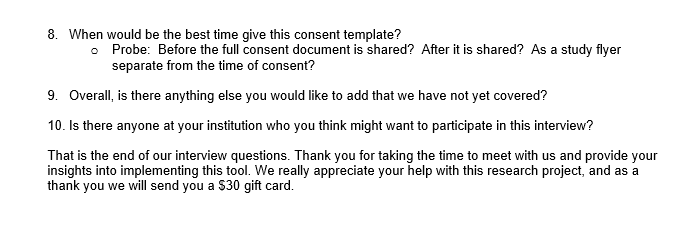

Supplement: Cooksey et al. supplementary material [file S2059866124006629sup001.docx]
